# Surface modification of polycaprolactone nanofibers through hydrolysis and aminolysis: a comparative study on structural characteristics, mechanical properties, and cellular performance

**DOI:** 10.1038/s41598-023-36563-w

**Published:** 2023-06-09

**Authors:** Raziye Yaseri, Milad Fadaie, Esmaeil Mirzaei, Hadi Samadian, Alireza Ebrahiminezhad

**Affiliations:** 1 Department of Medical Nanotechnology, School of Advanced Medical Sciences and Technologies, Shiraz University of Medical Sciences, Shiraz, Iran; 2 Nanomedicine and Nanobiology Research Center, Shiraz University of Medical Sciences, Shiraz, Iran; 3 Department of Tissue Engineering, School of Advanced Technologies in Medicine, Hamadan University of Medical Sciences, Hamadan, Iran; 4 Biotechnology Research Center, Shiraz University of Medical Sciences, Shiraz, Iran

**Keywords:** Biomaterials, Biomaterials - cells, Biomedical materials, Tissues, Nanomedicine

## Abstract

Hydrolysis and aminolysis are two main commonly used chemical methods for surface modification of hydrophobic tissue engineering scaffolds. The type of chemical reagents along with the concentration and treatment time are main factors that determine the effects of these methods on biomaterials. In the present study, electrospun poly (ℇ-caprolactone) (PCL) nanofibers were modified through hydrolysis and aminolysis. The applied chemical solutions for hydrolysis and aminolysis were NaOH (0.5–2 M) and hexamethylenediamine/isopropanol (HMD/IPA, 0.5–2 M) correspondingly. Three distinct incubation time points were predetermined for the hydrolysis and aminolysis treatments. According to the scanning electron microscopy results, morphological changes emerged only in the higher concentrations of hydrolysis solution (1 M and 2 M) and prolonged treatment duration (6 and 12 h). In contrast, aminolysis treatments induced slight changes in the morphological features of the electrospun PCL nanofibers. Even though surface hydrophilicity of PCL nanofibers was noticeably improved through the both methods, the resultant influence of hydrolysis was comparatively more considerable. As a general trend, both hydrolysis and aminolysis resulted in a moderate decline in the mechanical performance of PCL samples. Energy dispersive spectroscopy analysis indicated elemental changes after the hydrolysis and aminolysis treatments. However, X-ray diffraction, thermogravimetric analysis, and infrared spectroscopy results did not show noticeable alterations subsequent to the treatments. The fibroblast cells were well spread and exhibited a spindle-like shape on the both treated groups. Furthermore, according to the 3-(4,5-dimethylthiazol-2-yl)-2,5-diphenyltetrazolium bromide (MTT) assay, the surface treatment procedures ameliorated proliferative properties of PCL nanofibers. These findings represented that the modified PCL nanofibrous samples by hydrolysis and aminolysis treatments can be considered as the potentially favorable candidates for tissue engineering applications.

## Introduction

Tissue engineering is an interdisciplinary approach that aims to improve the repair and/or regeneration of injured tissues applying a combination of engineered scaffolds, cells and proper physicochemical and biochemical agents. Among these factors, scaffolds play a crucial role in the regeneration process by providing a potentially suitable substrate for the cells to adhere, proliferate, differentiate, migrate, and elicit the desired functions^1,2^.

Emerging of nanomaterials has revolutionized the tissue engineering approaches through their inherent capabilities for developing more desirable scaffolds. Nanostructures are innovative materials with precisely tailored characteristics which are applicable as scaffolds and drug delivery vehicles in tissue engineering applications. In recent years, a wide range of nanostructures have been developed and evaluated for tissue engineering applications^3–5^. Among them, electrospun nanofibers have been noticed unprecedentedly due to the some unique properties, such as high surface-to-volume ratio, mimicry of the native extracellular matrix (ECM) and potential of being fabricated from a wide range of natural, synthetic, and semi-synthetic polymers in different geometry, morphology, and architectures^6–9^. In addition, they are capable of being loaded, incorporated and functionalized by drugs and a wide variety of therapeutic agents during or after the fabrication process^10–14^.

The acquired nanofibrous scaffolds from the natural polymer sources have shown promising biocompatibility, rapid biodegradation rate, and favorable interactions with the cells. However, these structures usually suffer from the batch-to-batch variation of the source polymer as well as poor mechanical properties of the resultant scaffold^15,16^. Alternatively, nanofibers of some synthetic polymers (polyesters), such as poly-glycolic acid (PGA), poly-lactic acid (PLA) and poly (ε-caprolactone) (PCL) can be the beneficial candidates for tissue engineering purposes. For instance, the obtained nanofibers from PCL, as a semi-crystalline linear polyester, have a great potential to represent desirable mechanical performance along with the proper biocompatibility and biodegradation behavior^17,18^.

However, the hydrophobic nature and poor cell attachment profile of the PCL nanofibers are the main drawbacks that seriously hinder application of PCL nanofibrous scaffolds in the field of tissue engineering. It is clearly known that, poor surface wettability of the nanofibrous materials not only diminishes their bioactivity but also might induce cytotoxic effects^19^. Accordingly, PCL nanofibers are not capable to be an ideal scaffold unless their surface wettability being modified. Fortunately, these problems can be largely addressed by applying some distinct approaches such as blending or co-electrospinning with the bioactive agents or natural polymers, surface graft polymerization, plasma treatment and wet chemical modification methods^20–24^.

Blending or co-electrospinning with natural polymers is a widely used technique for the modification of PCL-based nanofibrous scaffolds. However, there are major concerns about the immiscibility of natural and synthetic polymers in routine solvents as well as potential inactivation of bioactive agents during the blending process^6,25,26^. In addition, some of the mostly used solvents on this approach have been known as the biologically hazardous materials^27^.

Surface graft polymerization of bioactive agents is a well-known approach to modify the surface of PCL nanofibers along with the induction of multi-functionalities to the nanofibrous membrane. However, prosperity of this method tightly hinge on the availability of dense and oriented surface functional groups on PCL nanofibers to interact with the bioactive agents. Therefore, this approach must be performed using effective surface conjugation/polymerization techniques which are not easily attainable^28,29^.

Plasma treatment is also a renowned approach based on a low-pressure gas process to activate the surface of PCL nanofibers. In this technique, various feed gases, such as oxygen, argon, nitrogen and carbon dioxide can be applied to introduce carboxylic acid, amine, and hydroxyl groups on the surface PCL nanofibers. The introduced surface functional groups can modify some properties of the PCL nanofibers by activating their surfaces^30,31^. However, as a major shortcoming, this modification technique only induces a superficial activation to the nanofibrous mats and the bulk of sample is not modified efficiently^32^.

Wet chemical modification is a facile, straightforward and efficient approach for the surface modification of PCL nanofibers. Ester groups (–COO–) within the microstructure of PCL nanofibers can be easily hydrolyzed to the hydroxyl and carboxylic acid groups through an alkaline treatment. It has been proven that, this modification can remarkably ameliorate the surface wettability as well as cellular behavior of the PCL nanofibrous mats. To exemplify, Chen et al. made an obvious enhancement in the spreading and attachment of 3T3 fibroblast cells cultured on surface modified PCL nanofibers. These PCL nanofibers which had been treated by 5 M NaOH solution for 3 h showed a highly improved surface wettability^7^. In another study, Park et al.^33^ represented that, osteoblasts have significantly better cell attachment and proliferation on the treated PCL nanofibers (1N NaOH for 1 h) compared to the pristine PCL nanofibers.

In addition, active amino groups can be introduced onto the PCL nanofibers through aminolysis reactions using the di-amine compounds. In this process, one amino group reacts with the ester group of PCL to form –CONH– (a covalent bond), while the other amino group of the compound dangles onto the surface of PCL nanofiber. The as-generated amino groups act as a linker and accelerate an attachment site for biomolecules and other bioactive agents onto the surface of PCL nanofibers. This capability can provide some valuable advantages for the PCL nanofibrous membrane like the enhancement of hydrophilicity and water uptake capacity. Additionally, the aminolyzed PCL samples might have superior biocompatibility thanks to the neutralized acidic byproducts generated during the degradation and more accessible active sites for bioconjugation of biological molecules and anchoring of the cells^34^.

**Figure 1:**
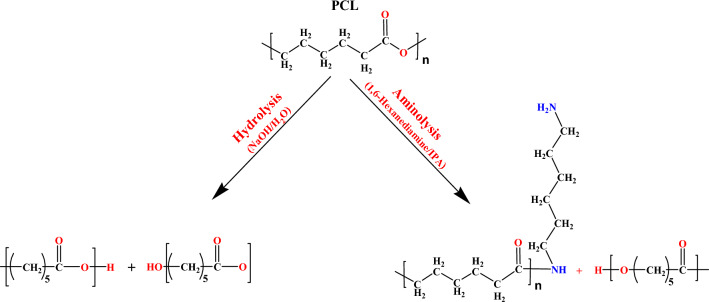
An illustration on molecular mechanism of the surface modifications (hydrolysis and aminolysis treatments) for PCL nanofibers.

In the current study, a range of hydrolyzed and aminolyzed samples were prepared and the potential effects of hydrolysis and aminolysis on the physical, mechanical, and cell compatibility of PCL electrospun nanofibers were comparatively investigated. Figure 1 illustrates a schematic on the molecular mechanism of chemical treatments have applied in this study^35–38^. The concentration of the reactive agents and the reaction time varied to compare the modifying effects of the treatment conditions on PCL nanofibers. For this purpose, neat PCL nanofibrous mat and the representatives for the treated samples were fully studied from the microstructural, mechanical and cellular behavior aspects.

## Results and discussion

### Morphological and structural traits

**Figure 2:**
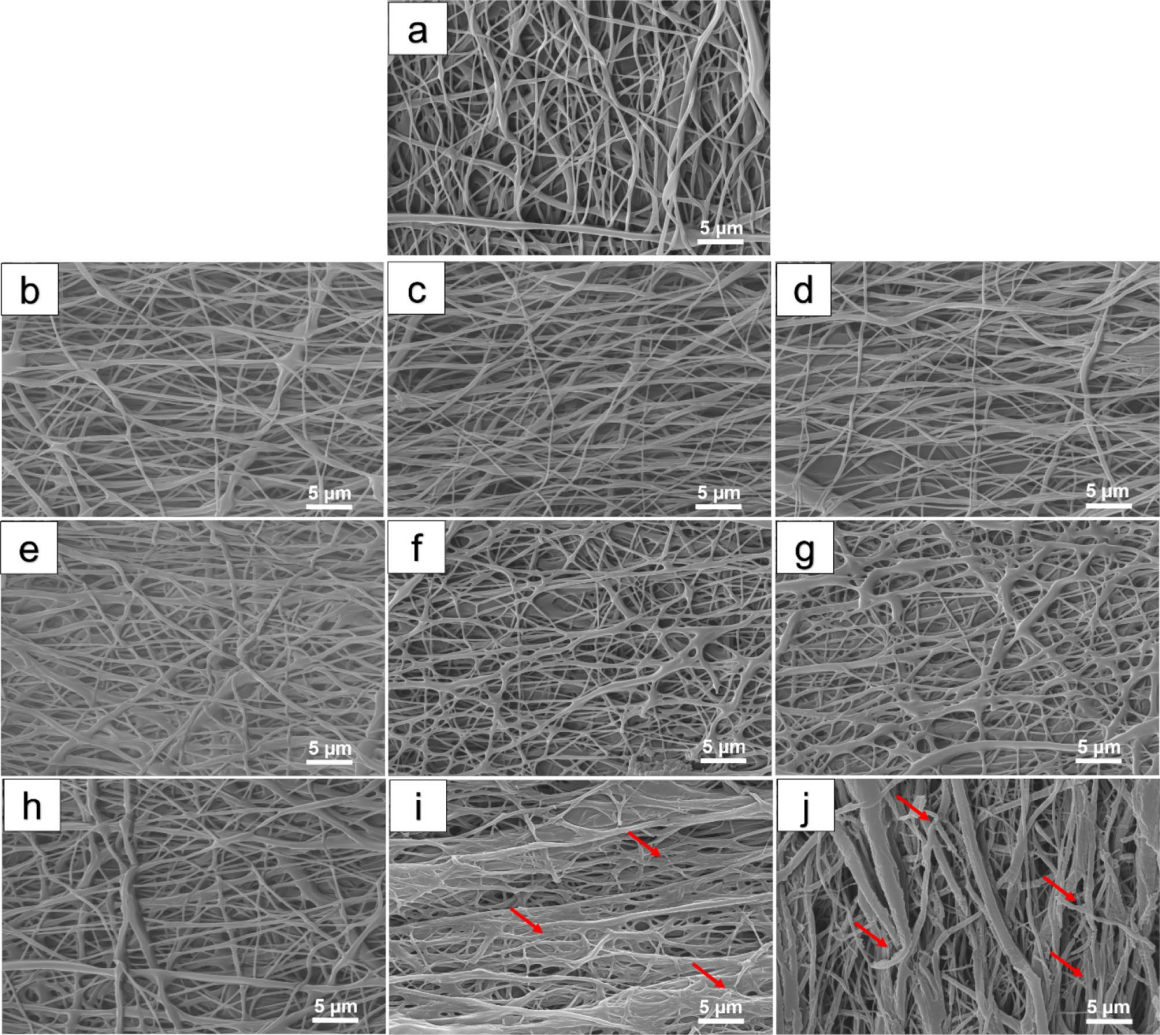
FESEM images of the PCL nanofibrous samples treated by different concentrations of NaOH solution and in various incubation time points (1, 6 and 12 h). (**a**) Pristine PCL, (**b**) S1, (**c**) S2, (**d**) S3, (**e**) S4, (**f**) S5, (**g**) S6, (**h**) S7, (**i**) S8, (**j**) S9. Red arrows indicate the structural defects and breakage of nanofibers.

**Figure 3:**
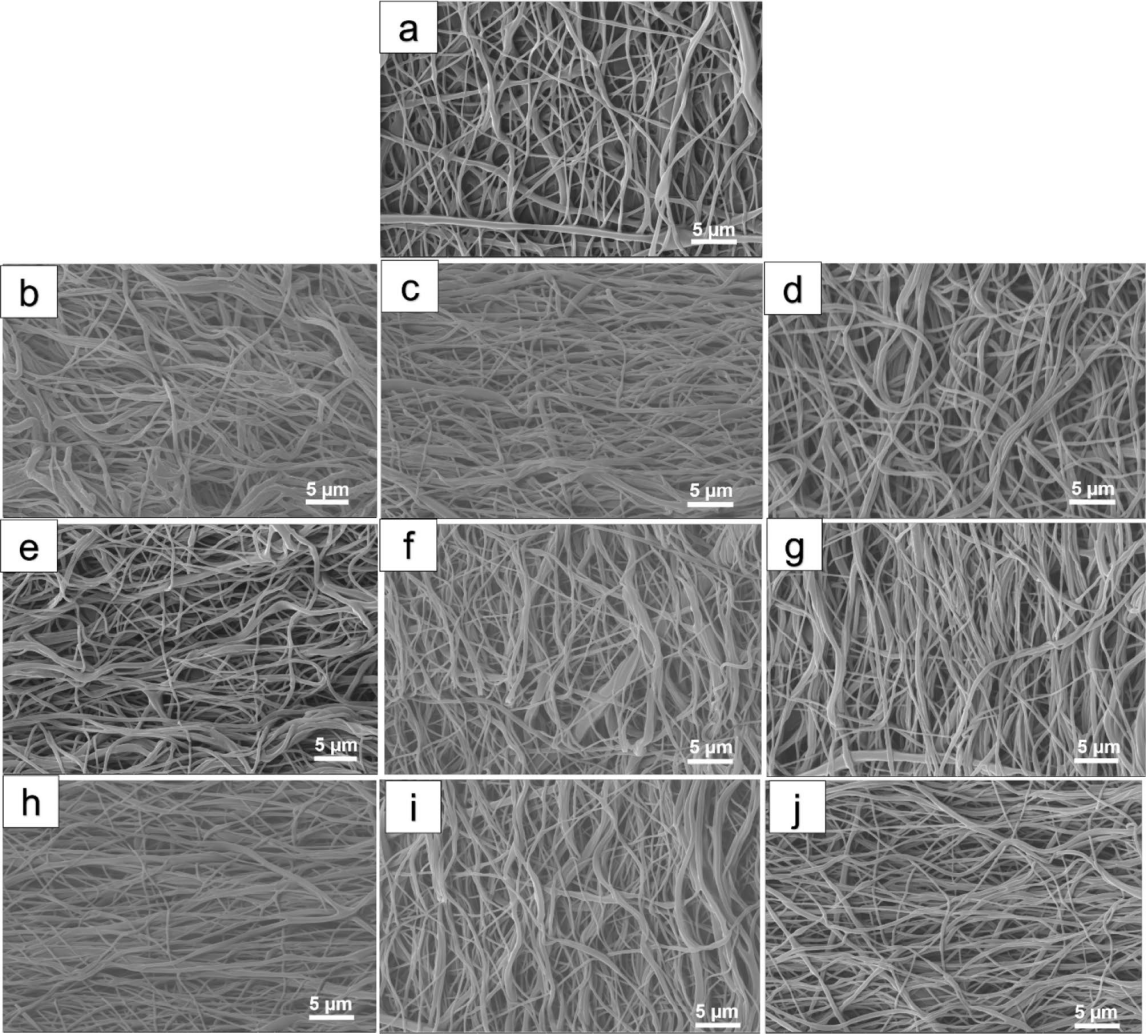
FESEM images of the PCL nanofibrous samples treated by different concentrations of HMD/IPA solution in various incubation time points (1, 12 and 24 h). (**a**) Pristine PCL, (**b**) S10, (**c**) S11, (**d**) S12, (**e**) S13, (**f**) S14, (**g**) S15, (**h**) S16, (**i**) S17, and (**j**) S18.

Field emission scanning electron micrographs of the neat and modified samples were utilized to investigate the structural and morphological properties of the PCL-based nanofibrous scaffolds. The obtained micrographs of the hydrolyzed and aminolyzed PCL samples are represented in Figs. 2 and 3, correspondingly. Since the electrospun PCL scaffold was the basic platform for the preparation of the modified samples, its morphological traits were required to be examined precisely. As expected from a properly-fabricated scaffold, PCL sample denotes three-dimensional porous structure organized from heterogeneous interconnected pores. In general, the PCL had a bead-free nanofibrous structure with smooth nanofibers. From the structural point of view, non-woven and randomly-oriented nanofibers could be clearly distinguished within the structure of PCL sample. According to the corresponding micrographs (Fig. 2a), PCL nanofibers indicate desirable surface morphology in respect of having a smooth and uniform framework. The porosity and pore size of PCL sample were examined using Image J software to quantitative investigation of the porous structure. The porosity and mean pore size of PCL sample were found to be around 43 ± 17% and 1512 ± 816 nm, respectively. Additionally, the average fiber diameter of PCL sample was calculated 305 ± 148 nm which was in consistent with the findings from previous studies^39^.

In the case of hydrolyzed samples, when the concentration of the solution was kept constant at 0.5 M, PCL nanofibers did not endure any drastic change in morphology by prolonging the treatment time (from 1 to 12 h). More precisely, not only did the smooth and uniform morphology of PCL nanofibers experience any considerable change, but also the mean fiber diameter and porosity percentage remained relatively constant. For example, for the S1 sample, the average fiber diameter (314 ± 143 nm) and porosity (41 ± 12%) were close to the measures obtained for PCL sample.

For 1 M NaOH, after 1-h treatment, the nanofibrous structure remained intact and defect-free (S4 sample). However, by increasing the incubation time to 6 and 12 h (S5 and S6 samples), flattened morphology appeared and the neighboring nanofibers were spatially packed. For instance, the resulting average diameter of nanofibers for S6 sample showed an obvious change in diameter to 402 ± 276 nm, but the porosity for this sample (S6) was found very similar to the PCL. When the concentration of NaOH was increased to 2 M and the treatment time was 1 h (S7 sample), no substantial changes in nanofibrous structure was observed. Nevertheless, when the immersion time gradually expanded to 6 and 12 h (S8 and S9 samples), enormous changes were observed on the surface of hydrolyzed nanofibers. In addition, the uniform and smooth structure of nanofibers were altered to a harsh and more heterogeneous structure. Moreover, some structural defects and breakage of nanofibers were induced to the surface of PCL as shown in Fig. 2i and j, red arrows. Based on more investigation, the porosity of nanofibers in the highest immersion time (S9 sample) revealed a drastic decrease to 36 ± 18%. Additionally, the average diameter nanofibers in the S9 sample was calculated at 381 ± 409 nm.

Overall, these observations implied that when the PCL is subjected to an extended time of higher concentration of hydrolysis solution, surface erosion of the nanofibers and subsequent change in fiber diameter may occur. Previous studies confirmed these findings. In a study by Yew et al.^23^, PCL nanofibers were exposed to different alkaline hydrolysis solutions for various durations. They found that slight erosion and rougher surface of fibers happened at the prolonged time of immersion in higher alkaline hydrolysis concentrations. Subsequently, the decrease in average thickness of PCL nanofibers membrane occurred in order to the peeling effect.

In terms of the introduction of amine groups onto the surface of PCL nanofiber, aminolysis was utilized and the effect of this treatment on the morphological structure of PCL nanofibrous scaffold was investigated (Fig. 3). The SEM images demonstrated slight changes in the morphology of scaffolds after aminolysis. There was no remarkable difference among treated scaffolds at various concentrations and times. In all aminolyzed samples, the smooth surface of PCL nanofibrous scaffold turned into a wrinkle-like surface and became partly rough. In addition, the aminolyzed nanofibers tended to form fused nanofibers. Furthermore, the diameter of aminolyzed samples was slightly increased in comparison with pristine-PCL. In order to elucidate, the analysis of the S18 sample showed an increase in mean diameter, 350 ± 190 nm, this was possibly due to fused nanofibers. Furthermore, the porosity of all aminolyzed PCL nanofibers was diminished and reached to the values between 24 ± 10% and 37 ± 20%. These data in comparison with pristine-PCL (the porosity of 41 ± 12%) indicate a more compact fibrous density.

Generally, it is depicted in Fig. 3 that the surface of samples partly had changed, but these changes are not very harsh, as in previous literature^40^. Another study by Amoures de Sousa et al.^41^ confirmed that there was no alteration on the morphology of PCL nanofibers after the aminolysis process. In another study, Krithica et al., used 10% HMD/IPA for 12 h at 37 °C to introduce nitrogen-containing groups on the nanofibers. They reported that the treatment did not induce any adverse effects on the morphology of the nanofibers^42^.

The current work was employed to assess the effect of two types of wet chemical treatment (aminolysis and hydrolysis) on the properties of PCL scaffold. Our findings from SEM micrographs of functionalized samples regarded that both chemical treatments had changed the morphological structure of the PCL. As discussed above, aminolysis solution with various concentrations and duration had a similar partial effect on PCL sample. On the contrary, these changes for the hydrolyzed sample were ascribed to the concentration of the treatment solution and time span. In brief, the morphology of the PCL had more changes by dipping in an alkaline hydrolysis solution in comparison with the aminolysis solution.

### Surface wettability

**Figure 4:**
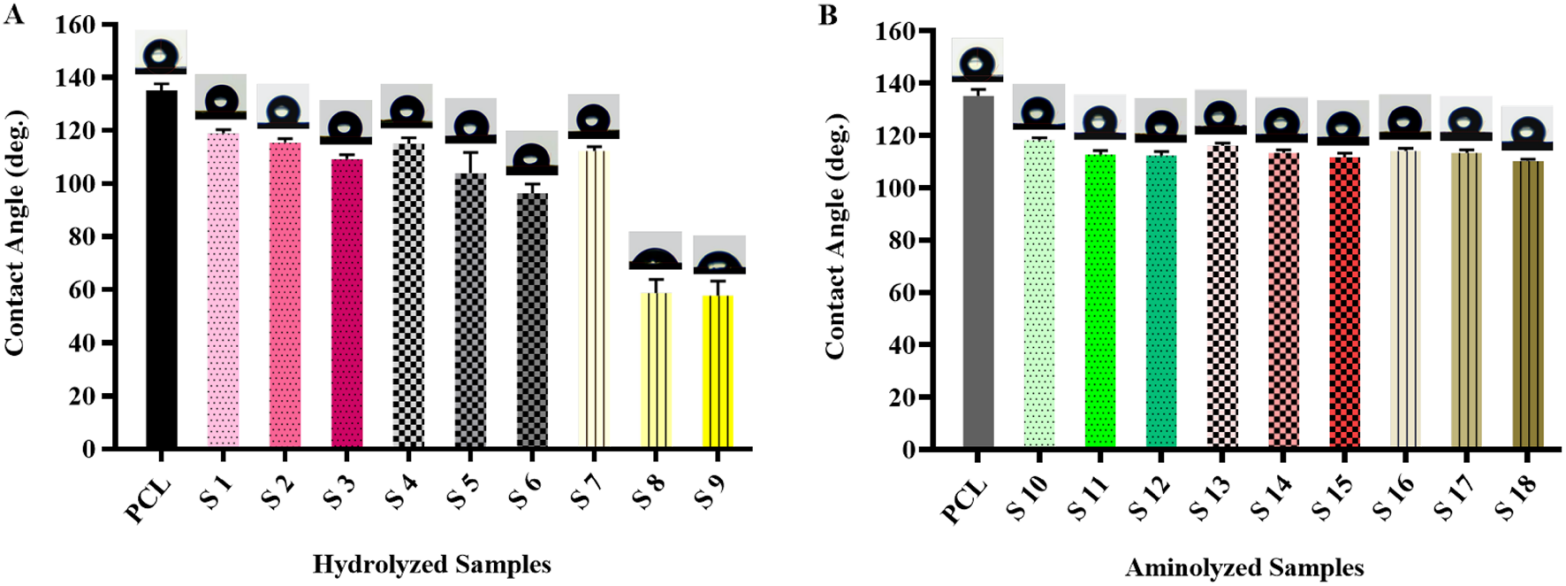
Water contact angle values of the (**A**) hydrolyzed PCL nanofibrous samples and (**B**) aminolyzed PCL nanofibrous samples.

As a substantial criterion for the evaluation of hydrolysis and aminolysis treatments, the surface wettability of treated PCL nanofibrous mats was investigated and compared with pristine PCL. In this respect, water contact angle (WCA) analysis in a specified time point (sec 5) was applied to make a quantitative comparison (Fig. 4A and B). The WCA for pristine PCL nanofibrous sample was measured 135.09° ± 2.44° that was an obvious indicative for its hydrophobic nature. It has been already proven that, to obtain a promising cellular behavior, the hydrophobicity of PCL sample must be ameliorated to an optimal level^43^. Principally, a balanced rate of hydrophilicity on the surface provide a great potential in developing more anchoring sites for the initial adhesion of cells. A proper initial cell adhesion can result in superior cellular colonization on the surface of scaffold which makes it more compatible for spreading, infiltration and proliferation of a variety of cells^44^.

As a general trend, surface hydrophilicity of the PCL nanofibrous membrane was improved subsequent to the wet chemical treatments. Clearly, by increasing the concentration of treatment solutions and prolonging the incubation time, surface wettability of the PCL sample enhanced in both hydrolysis and aminolysis procedures. However, the amount of this improvement was comparatively different. More precisely, the extreme hydrolysis conditions resulted in a more intense decrease in WCA of PCL comparing with the extremely aminolyzed samples. It was clearly distinguishable in case of S9 and S18 samples which had 57.56° ± 5.61 and 110.41° ± 0.68 WCA respectively.

The case is that, induction of active surface functional groups (–OH, –COOH, –NH_2_) can greatly increase the hydrophilicity of nanofibers. Not to mention that highly functionalized surface have superior capability for uptake and retention of water molecules. Additionally, a considerable improvement in hydrophilicity of hydrolyzed nanofibers compared to the aminolyzed nanofibers might be attributed to the higher cleavage reaction that is terminates in greater extent for hydroxyl and carboxyl groups. The overall results verified that treatment methods effectively enhanced the wettability of samples that is in line with the some previous studies^33,45–47^.

### Mechanical performance

**Figure 5:**
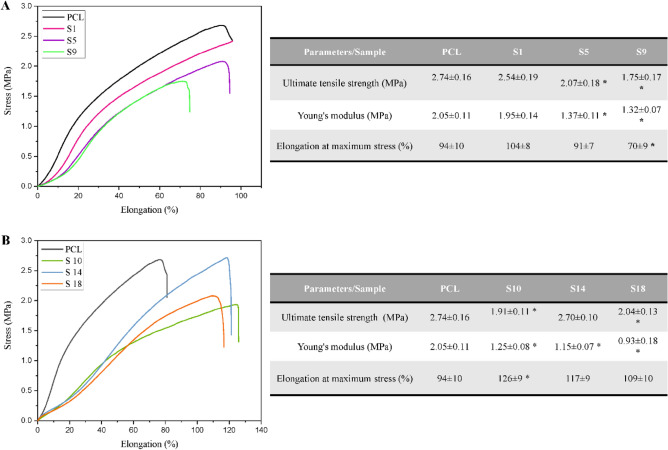
Stress–Strain curves and table of the corresponding mechanical parameters for the (**A**) hydrolyzed PCL nanofibrous samples and (**B**) aminolyzed nanofibrous samples. * Indicates statistically significant difference in comparison with the PCL sample (p < 0.05).

An ideal nanofibrous scaffold should have desirable mechanical properties for applying in tissue engineering applications^48^. From this, it was crucial to evaluate the mechanical behavior of PCL scaffolds. To this purpose, PCL-based nanofibrous samples were subjected to an evaluation method based on uniaxial tensile testing up to the structure failure. The ultimate tensile strength (UTS), Young’s modulus and elongation at maximum stress were measured from the obtained stress–strain curves for all samples. To simplify the comparisons and appointing an analyzable trend, only chosen samples for hydrolysis (S1, S5, S9) and aminolysis (S10, S14, S18) are further represented and discussed. The results for opted samples are summarized in Fig. 5 and the results for other samples are represented in the Supplementary data. It is worth mentioning that, variations in the results of replications for each sample was inevitable. It could be attributed to the slight inescapable changes in electrospinning process as well as delicate errors in the procedure of mechanical testing. Accordingly, in order to minimize the impact of these factors, fabrication process and testing procedure were tried to be supervised strictly to reduce the potential errors. In addition, the obtained data from replications of each sample were averaged when the deviated ones were omitted.

As a general trend, the lower concentration of NaOH treatment solution along with the shorter incubation time do not make significant impact on the mechanical performance of PCL nanofibrous sample (S1). On the contrary, by increasing the concentration of treatment solution and incubation time (S5 and S9), the mechanical parameters are influenced significantly. To exemplify, the tensile strength of pristine PCL (2.73 ± 0.16 MPa) significantly decreased to 2.07 ± 0.18 and 1.75 ± 0.17 MPa for S5 and S9 samples, respectively (p < 0.05). The same trend was observed for Young’s modulus when pristine PCL experienced a significant decline from 2.04 ± 0.11 MPa to 1.36 ± 0.11 and 1.32 ± 0.07 MPa for S5 and S9 samples correspondingly. However, in terms of percentage of strain, significant variation from the PCL (94 ± 10%) was merely detected in S9 curve (70.6 ± 9%).

The above-mentioned variations in mechanical behavior of samples can be largely justified by the developed changes in their structural properties which can be seen in SEM micrographs. For instance, the fact that, structure of S1 sample had been mainly preserved compared to pristine PCL, can be the major reason for making no significant changes in the mechanical properties of this sample. However, in the case of S5 sample, PCL nanofibers were experienced a degree of flattening, packing with neighboring nanofibers and some breakages in their structure which can lead to a slight but significant decrement in the mechanical stability. Similarly, the mechanical performance of S9 sample had been negatively influenced by the enormous changes in structural properties of PCL nanofibrous network. In this case, burden damages to the surface of PCL nanofibers along with the clear disruptions through interconnected nanofibrous structure of sample has contributed to undeniable weakening of mechanical behavior. It is worth noting that, the obtained mechanical findings for the hydrolyzed samples were largely in consistent with the results of study by Sonthaya Chaiarwut et al.^48^.

Figure 5B exhibits the effect of various aminolysis conditions on the mechanical properties of PCL-based nanofibrous samples. The quantitative data for PCL, S10, S14, and S18 samples were obtained from the corresponding stress–strain curves. In general, aminolyzed samples had relatively lower tensile strength comparing with the PCL. In terms of Young’s modulus, a distinct downward trend, from 2.04 ± 0.11 MPa for pristine PCL to 0.93 ± 0.18 MPa for S18, could be detected. The variations of Young’s modulus of samples were statistically significant when compared to the PCL. Nonetheless, the elongation of aminolyzed PCL-based samples increased to 126 ± 9, 117 ± 9 and 109 ± 10% for S10, S14 and S18 respectively. As expected, rigidity of was evidently reduced when PCL-based samples were modified through the aminolysis conditions that could be ascribed by distinguishable changes in the orientation of random PCL nanofibers towards a more orderly network. This might be the contributing factor for enhancement of elasticity as well as reduction of tensile strength (strain) in the aminolyzed samples. In the case of remarkable decrease in Young’s modulus of samples, it is assumed that, aminolysis treatments can potentially affect structure of PCL nanofibrous network in a deeper level comparing with the hydrolysis. In a previous study by Zhu et al.^34^ HMD/IPA treatment solution was used for aminolysis of PCL membrane to introduce free NH_2_ groups as the active sites for conjugating gelatin, chitosan, or collagen. They realized that the aminolysis reaction could penetrate as deep as 50 µm of the membrane; as a result, the Young’s modulus of samples were remarkably diminished. In another study, Toledo et al.^49^ showed that decrement of Young’s modulus in PCL nanofibrous samples is inevitable after aminolysis treatment by diamines. In addition, they found that aminolysis by HMD had more drastic effects on Young’s modulus rather than the other diamines.

### Surface functional groups

**Figure 6:**
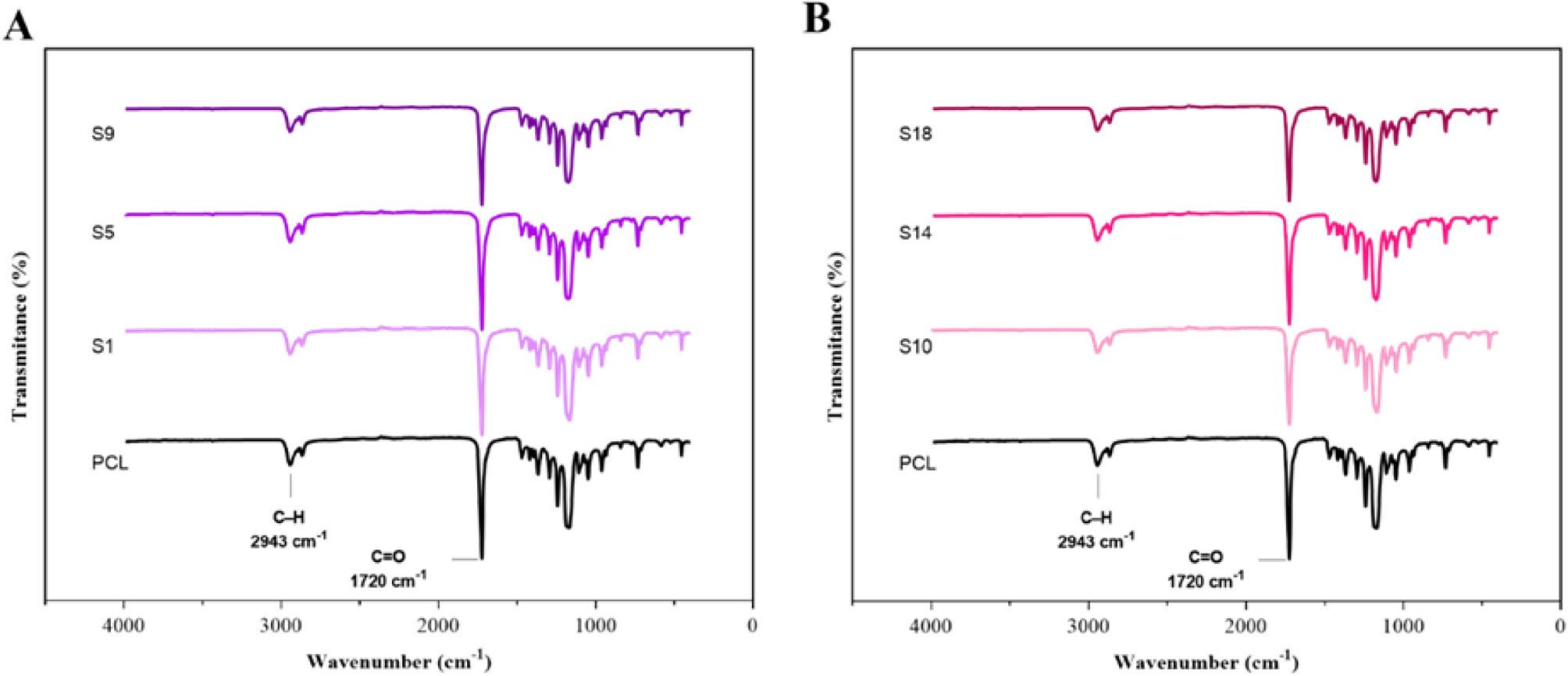
FTIR spectra of the representatives from (**A**) hydrolyzed PCL nanofibrous samples and (**B**) aminolyzed nanofibrous samples.

Attenuated total reflectance Fourier transform infrared (ATR–FTIR) spectrometry analysis was conducted to identify subjected functional groups on the modified samples through hydrolysis and aminolysis. Figure 6 illustrates the results of the neat and modified samples. In brief, during hydrolysis treatment, hydroxide ions, formed from dissolving of NaOH compound in the water, attack the carbonyl group of the PCL. Following this, hydroxyl and carboxyl end groups as shown in Fig. 1 are formed^50,51^. In addition, in a similar reaction in aminolysis, a diamine molecule breaks the ester bond in the PCL and attaches to it, resulting in amide and hydroxyl groups formation^50–52^. In the case of PCL, all the bending and stretching vibrations are found to be well-matched with the reported values; accordingly, the characteristic peaks are located at 1720 cm^−1^ and 2943 cm^−1^ which attributed to carbonyl (C=O) and C–H groups, respectively. Although the appearance of amide II (–NH) peak at 1560 cm^−1^ and hydroxyl (− OH) peak around 3200 cm^−1^ for aminolyzed samples and hydroxyl group around 3200 cm^−1^ for hydrolyzed samples were expected, no change in the spectra was observed compared to the neat PCL^39,49,53,54^. This may be due to the inadequate amount of functional amide and hydroyl groups to be detected by the ATR-FTIR instrument (depth ~ 1–2 µm)^49,53,55^.

### Thermogravimetric analysis

**Figure 7:**
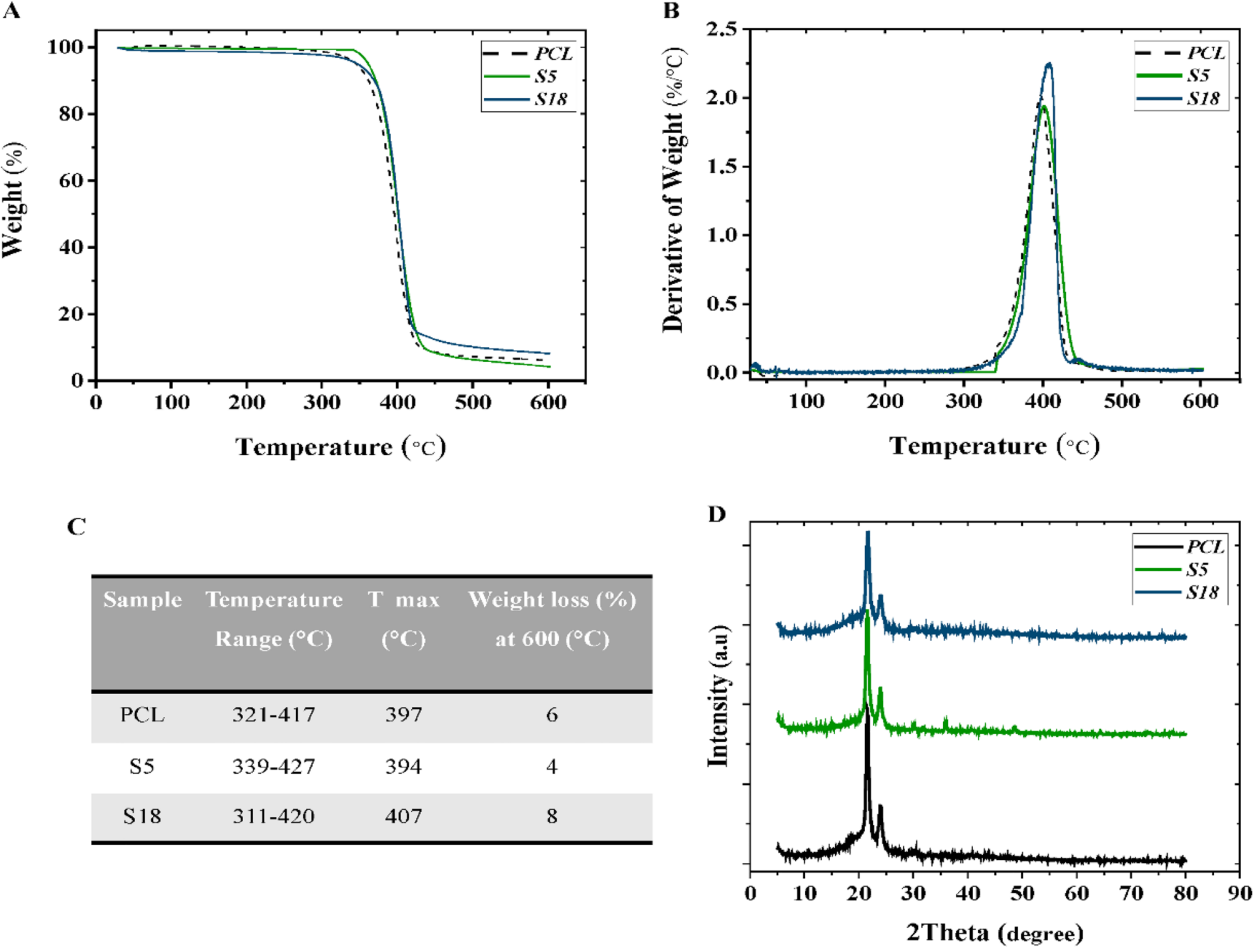
(**A**) TGA thermograms, (**B**) DTGA thermograms, (**C**) table of decomposition parameters, and (**D**) XRD pattern of PCL, hydrolyzed PCL (S5) and aminolyzed PCL (S18).

The thermal analysis was conducted to evaluate the thermal stability and decomposition behavior of PCL and modified nanofibers. Figure 7A–C indicate the results of TGA and DTGA of PCL as a decent sample, S5 and S18 samples as sound samples. In general, it is a known fact that PCL consists of single-stage decomposition with a maximum decomposition temperature (T_max_) of about 400 °C. As can be seen in the Figure, all samples (PCL, S5 and S18) denoted single-stage decomposition with an initial weight loss of 1–5% that can be attributed to the removal of residual moisture. Additionally, all samples started to decompose around 360 °C due to the pyrolysis of polyester chains and continued to the maximum decay at T_max_ of 397 °C, 394 °C and 407 °C that is belonged to PCL, S5, and S18 samples in order. According to these results, surface modifications did not deteriorate the thermal behavior of samples, which follows previous studies^53,56^.

### XRD patterns

The crystalline nature of PCL, S5, and S18 samples was investigated using XRD analysis, and the results are presented in Fig. 7D. On the whole, the crystallinity of PCL is recognized by two diffractive peaks that are related to the (110) and (200) planes of the orthorhombic crystal structure of PCL that could be observed at Bragg angles of 2θ = 23.6° and 21.3°^57,58^. According to the XRD graph, hydrolysis and aminolysis treatment do not affect the semi-crystalline structure of PCL. This result is in consistent with the prior literature^59^.

The XRD results along with TGA and FTIR results imply that hydrolysis and aminolysis do not affect the bulk of PCL nanofibers. The XRD, TGA, and FTIR results of hydrolyzed and aminolyzed PCL did not show any noticeable difference in comparison with pristine PCL. Therefore, the changes in morphology, mechanical properties, and hydrophilicity of nanofibers are the result of surface modification.

### Elemental analysis

Energy dispersive X-ray spectroscopy (EDAX) was utilized to determine the amount of elements on PCL and modified samples. In this analysis, elemental compositions including carbon (C), oxygen (O), and nitrogen (N), were measured and shown in Table 1. PCL has a certain amount of elements that could be changed under various conditions. Hence, hydrolysis and aminolysis treatment could change the type and quantity of elements on the surface of the PCL. As shown in Table 1, by increasing the duration of treatment and concentration of both hydrolysis and aminolysis solutions, a descending trend in the percentage of C and ascending trend in the percentage of O is seen. These could prove the formation of functional groups on the surface of PCL nanofiber. Additionally, after aminolysis, the quantity of N increased due to the amide bond on the surface of the PCL^37,60^.

**Table 1:** Percentage of the elements on PCL and representatives of the modified samples.

Samples/element	C		O		N	
	Weight %	Atomic %	Weight %	Atomic %	Weight %	Atomic %
PCL	67.91	73.82	32.08	26.18	0.01	0.01
S1	54.48	61.45	45.52	38.55	–	–
S5	54.30	61.28	45.70	38.72	–	–
S9	52.39	59.44	47.61	40.56	–	–
S10	60.84	67.36	38.50	32.01	0.66	0.63
S14	58.48	65.07	39.43	32.93	2.10	2.00
S18	56.94	63.47	38.84	32.50	4.21	4.03

### In vitro studies

**Figure 8:**
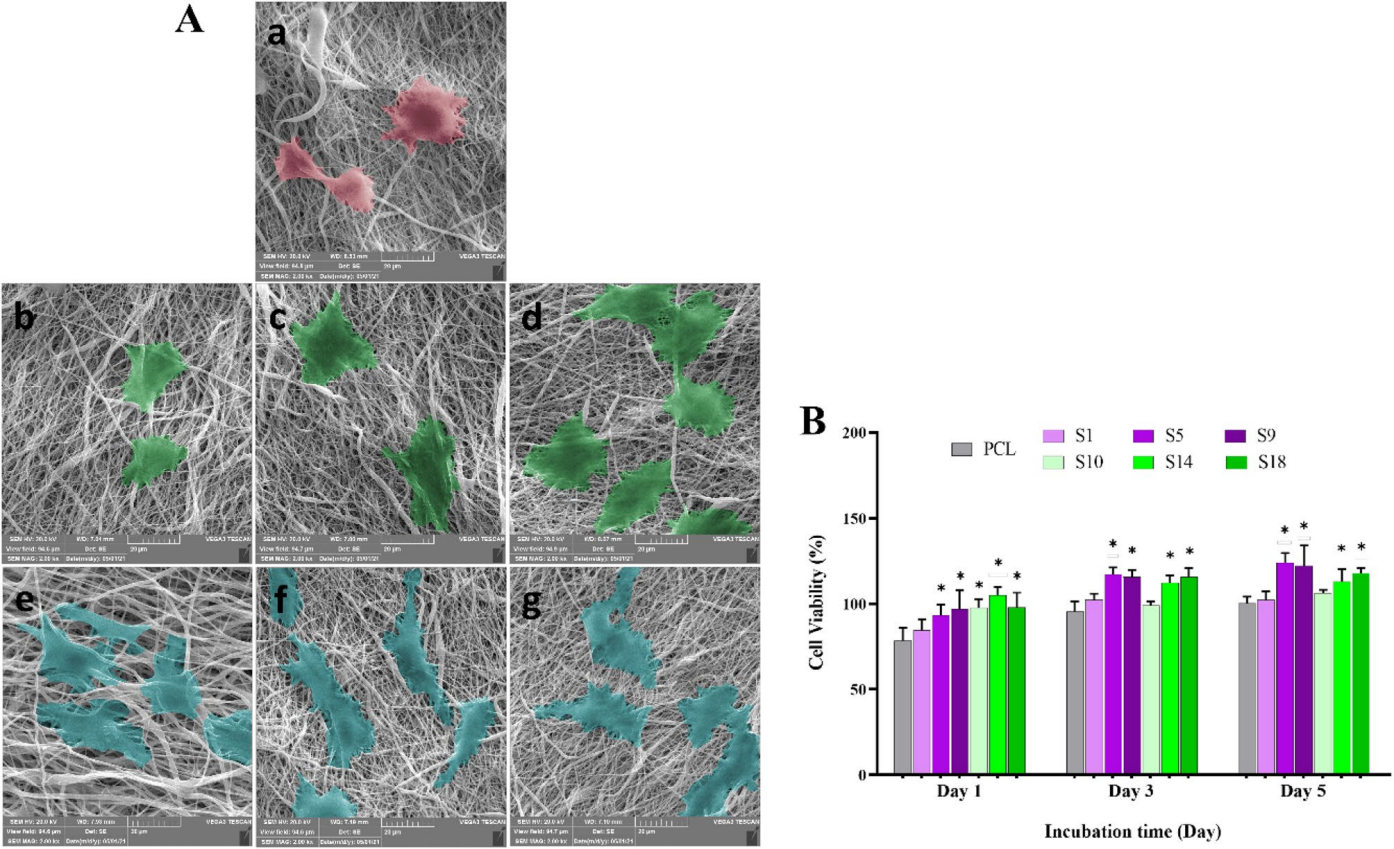
(**A**) SEM micrographs of the L929 fibroblasts cultured on PCL-based nanofibrous samples; (a) PCL, (b) S1, (c) S5, (d) S9, (e) S10, (f) S14, (g) S18. (**B**) Percentage of cell viability (relative proliferation) of the cultured L929 cells on pristine PCL, hydrolyzed samples (S1, S5, S9) and aminolyzed samples (S10, S14, S18) after 1, 3, and 5 days. *Indicates a statistically significant difference in comparison with the PCL sample (p < 0.05).

The viability of L929 fibroblast cells on the fabricated PCL nanofibers was evaluated using the MTT assay and the viability bar chart is shown in Fig. 8. Accordingly, the L929 cells were cultured on the PCL and modified samples to investigate cytocompatibility and cytotoxicity. The obtained results denoted that the proliferation of the cells on the PCL nanofiber was lower than the control group (cell culture plate) at each time point (1, 3, and 5 days). It should be noted that the viability of control group was considered 100% and the treat groups was compare with it. On the other hand, the surface treatments induced proliferative effects and made PCL nanofibers more favorable for cell proliferation. In comparison with PCL, the S5, S9, S14, and S18 samples showed significantly higher cell viability in all culture times (p < 0.05). There was no significant difference among S5, S9, S14, and S18 samples in each time points. These results imply that moderate hydrolysis and aminolysis (sample S5 and S14, respectively) are enough to enhance cell viability and proliferation on the PCL nanofibrous scaffold. Increasing the concentration of the treatment solutions and the duration of the treatment did not lead to increase in cell proliferation.

The significant cell proliferation in modified samples could be due to oxygen-containing and amino groups on the surface of nanofibers through the modifying process^61^. The presence of functional groups on the PCL nanofibers not only improved the hydrophilicity, but also provided active sites for biomolecule adsorption, cell attachment, and subsequently enhanced cell proliferation. The observed cell proliferation pattern is in agreement with previous studies. Zhu et al. reported that the aminolysis of PCL nanofibers promoted biomacromolecule immobilization and cell proliferation^34^. In another study, Mattanavee et al. confirmed the effectiveness of the introduced amine group in immobilizing biomolecules on the surface of PCL scaffold^62^.

Scanning Electron Microscopy (SEM, TESCAN-Vega3, Czech Republic) was utilized to assess the morphology of the adherent fibroblast cells on the PCL nanofibers. As a general rule, there is a close correlation between hydrophilicity and the shape of adherent cells^63^. In addition to surface wettability, the type of cells plays a crucial part in responding to the scaffolds. L929 fibroblasts have a tendency to spread on a rough surface rather than a smooth one^64^. However, the uniform diameter of PCL nanofiber advocates a proper scaffold for the proliferation of cells but the hydrophobic surface of PCL is not favorable for cell attachment and spreading^65^. Hence, the chemical treatment is an efficient way to prepare a highly desirable surface for cell interaction by inducing ideal surface functional groups (–OH, –COOH, –NH_2_). As shown in Fig. 8A, the fibroblast cells illustrate a spherical shape on the PCL, which can be due to the hydrophobic nature of the PCL. In contrast, the morphology of the cells on the surface-modified PCL nanofibers was spindle-like shaped, indicating that the cells exhibited their native morphology on the modified nanofibers. Furthermore, in hydrolysis and aminolysis treatments with time and concentration increment, the fibroblast cells widely expand concerning the presence of more functional groups. These observations indicate that both surface treatments favored nanofibers’ surface for the cell attachment and adaptation, which agree with SEM, WCA and EDAX results.

**Figure 9:**
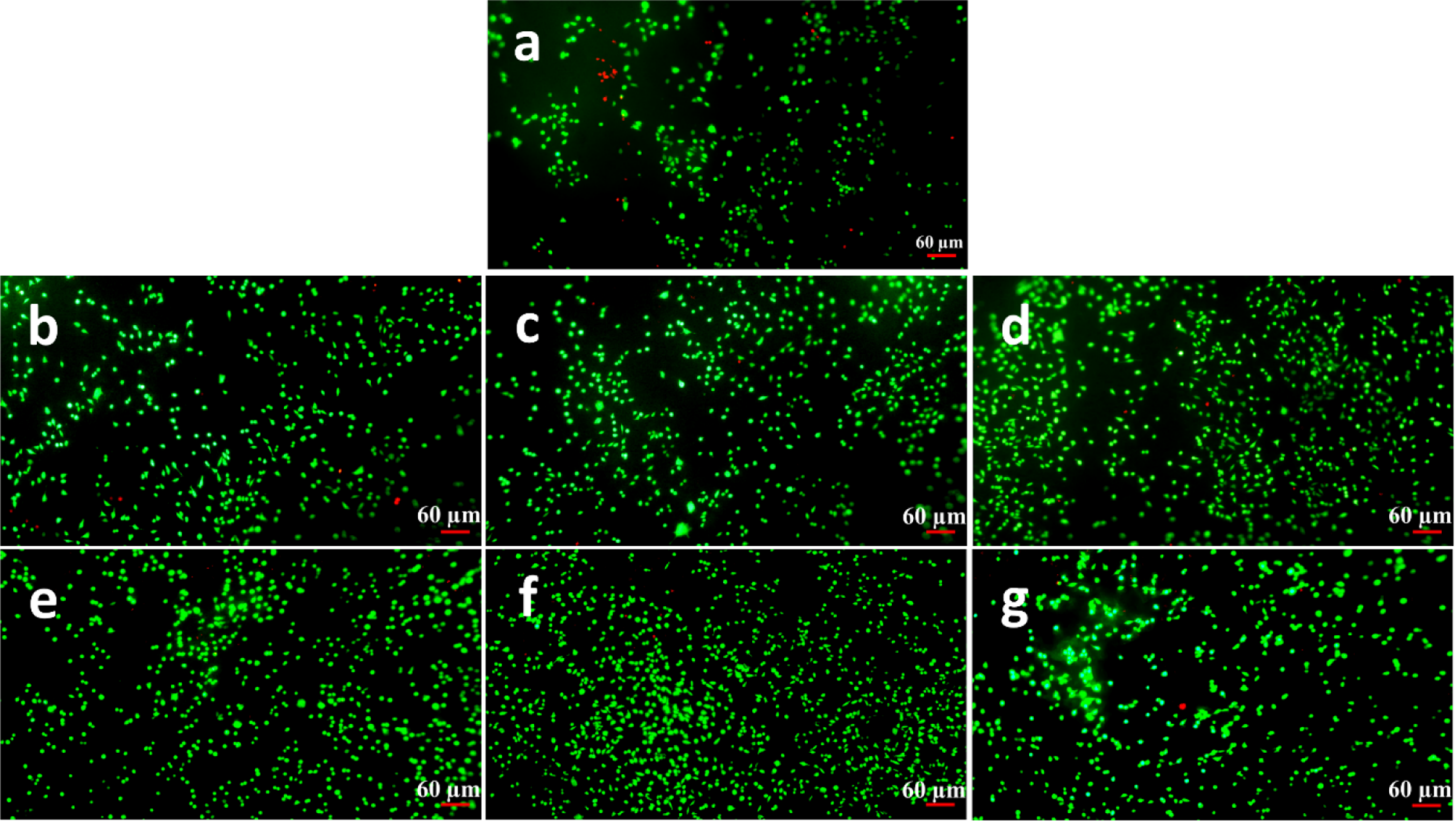
Fluorescence images of the L929 fibroblast cells cultured on the samples at the day 1 post culture; (**a**) PCL, (**b**) S1, (**c**) S5, (**d**) S9, (**e**) S10, (**f**) S14, (**g**) S18.

**Figure 10:**
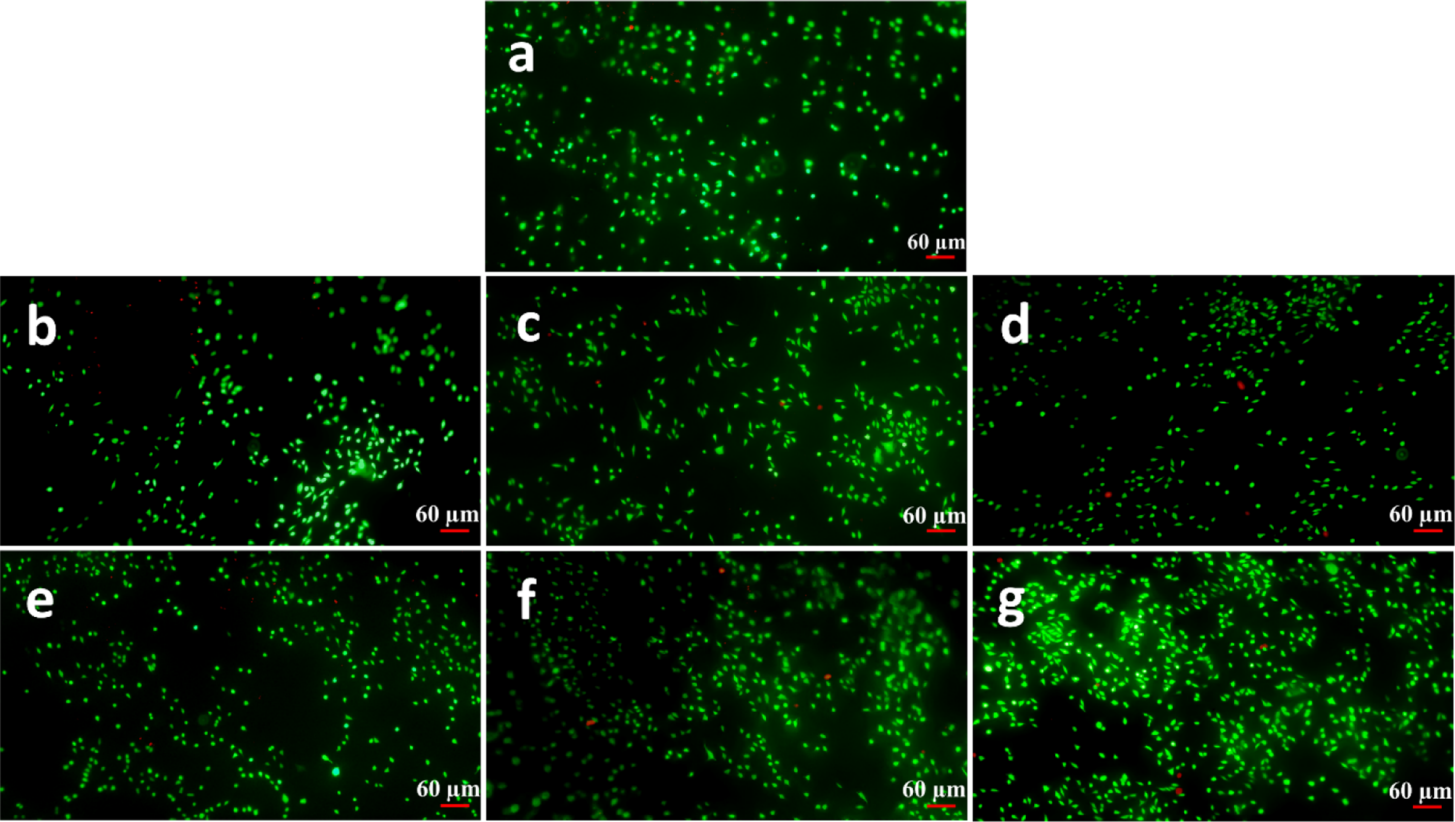
Fluorescence images of the L929 fibroblast cells cultured on the samples at the day 3 post culture; (**a**) PCL, (**b**) S1, (**c**) S5, (**d**) S9, (**e**) S10, (**f**) S14, (**g**) S18.

**Figure 11:**
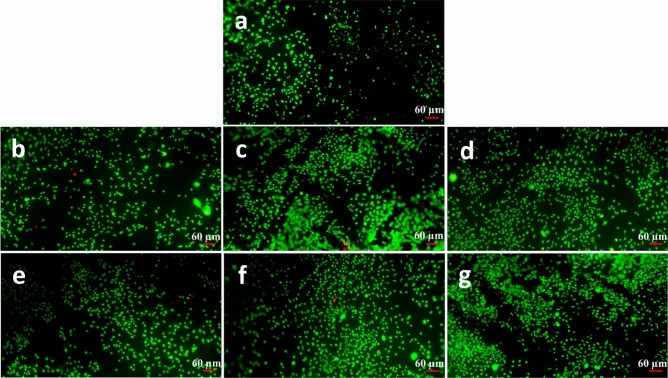
Fluorescence images of the L929 fibroblast cells cultured on the samples at the day 5 post culture; (**a**) PCL, (**b**) S1, (**c**) S5, (**d**) S9, (**e**) S10, (**f**) S14, (**g**) S18.

The Live/dead assay is an analyzing method to observe viable and dead cells and their partial morphology based on the fluorescent staining technique. Figures 9, 10, and 11 exhibit alive cells on the PCL and modified nanofibers at different time points (1, 3, and 5 days). Based on qualitative and visual observations, the density of live cells on the PCL was undeniably fewer than modified samples after 5 days due to the hydrophobicity of the PCL. In addition, increasing the cell number on modified samples is regarding the existence of a large number of functional groups on them^33,40^. Moreover, the cells cultured on the treated PCL nanofibers exhibited spindle-shaped and extended morphology corresponding to the SEM images. Overall, the beneficial effects of chemical treatments outweigh their detrimental effect.

## Conclusion

In the current study, surface modification of PCL nanofibers through hydrolysis and aminolysis treatment procedures was performed to develop a better platform for tissue engineering applications. To make an extensive insight on the influence of chemical treatments, pristine PCL sample and the surface-modified samples were thoroughly characterized and compared. According to the characterization findings, both aminolysis and hydrolysis mainly alter the surface properties of PCL nanofibers without any negative effects on their bulk properties. More precisely, in the severe cases, chemical treatments resulted in minor morphological changes and moderate decrement in mechanical performance. However, a tangible increase in the hydrophilicity of PCL nanofibrous mats were distinguishable in the most samples. Clearly, higher concentration of hydrolysis solution and longer incubation time lead to better enhancement of hydrophilicity in price of a relative bulk weakening. However, this trend was less sensible for aminolyzed samples. Notwithstanding, the microstructural features and mechanical performance of the opted samples were acceptable for some tissue engineering applications.

The obtained results from in vitro studies indicated that the surface modifications of PCL nanofibers not only induce any cytotoxic effect, but also provide an ideal surface for cell attachment, spreading and proliferation. The cultured L929 cells mainly exhibited their native fibroblastic morphology on the treated samples having multiple interaction sites with the hydrolyzed and aminolyzed PCL nanofibers. According to these findings, the hydrolyzed and aminolyzed PCL nanofibrous membranes, under particular conditions, could be served as potential candidates for tissue engineering scaffolds.

## Materials and methods

### Materials

PCL (Mw = 80,000 g mol^−1^), NaOH, and NaCl were purchased from Sigma-Aldrich (St. Louis MO, USA). Chloroform, *N*, *N*-Dimethylformamide (DMF), dimethyl sulfoxide (DMSO), and isopropanol were purchased from Merck (Darmstadt, Germany). MTT powder was acquired from Sigma Aldrich (Darmstadt, Germany). DMEM/F-12 cell culture medium, Fetal Bovine Serum (FBS), Penicillin–Streptomycin (Pen-Strep), and Trypsin- EDTA were obtained from Gibco (Thermo Fisher Scientific, Dreieich, Germany).

### Fabrication of the electrospun PCL mats

**Figure 12:**
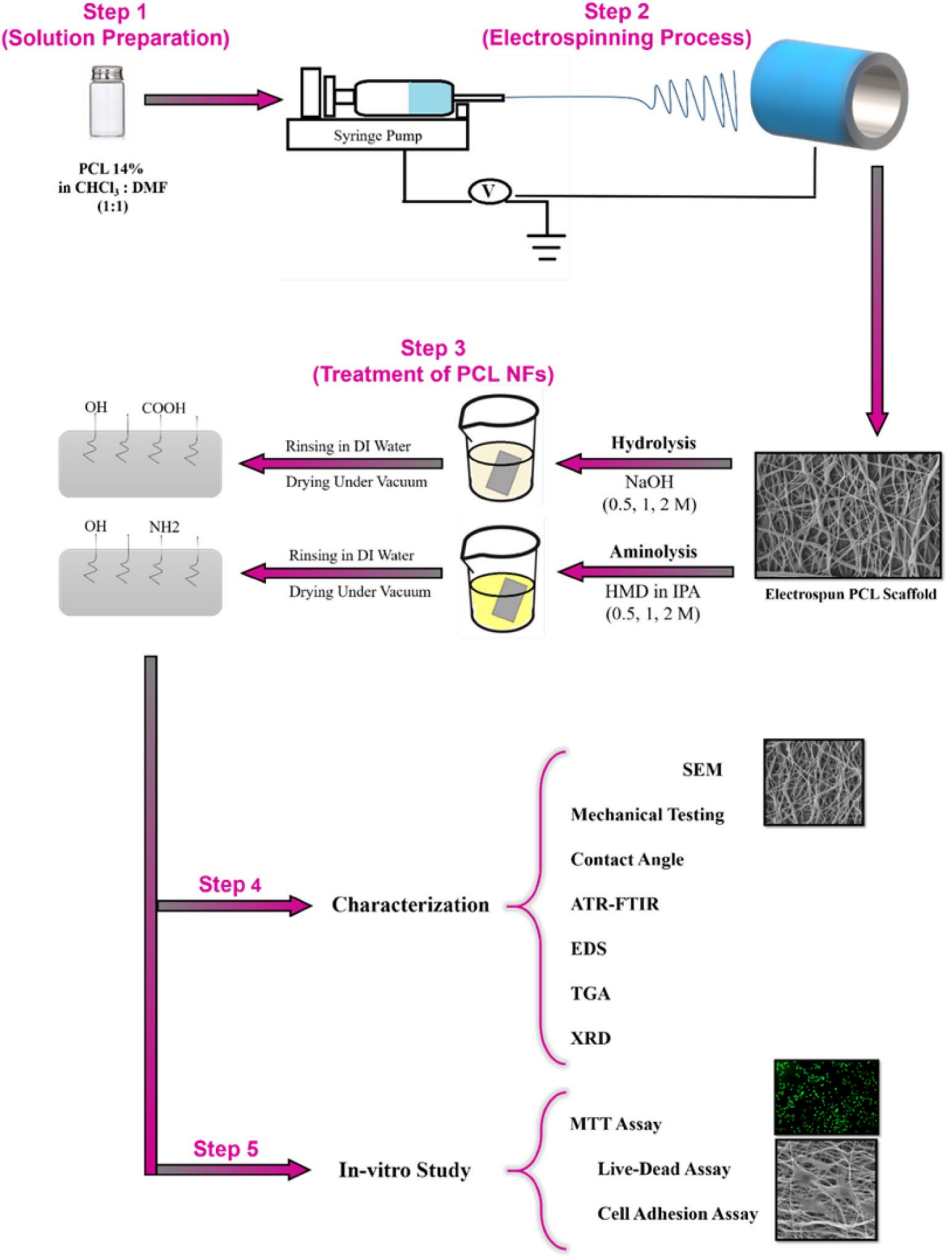
An overview on the fabrication process, chemical treatment procedures, characterizations and in-vitro studies.

PCL nanofibers were fabricated using a commercial electrospinning apparatus (Fanavaran Nano Meghyas Ltd., Co., Tehran, Iran). A proper amount of PCL was dissolved in a solvent mixture of DMF/Chloroform (50/50) to obtain the final concentration of 14 wt.%. The polymeric solution was stirred on a magnetic stirrer for 16 h to obtain a clear and homogenous solution. The obtained solution was loaded into a 5 mL syringe equipped with a 20-gauge blunt needle. The feeding rate, applied voltage, and nozzle-to-collector distance were set at 1.5 mL/h, 10 kV, and 140 mm respectively to collect the nanofibrous mat on the collector covered by aluminum foil. The obtained electrospun PCL mats were prepared for surface modification treatment procedures and further characterizations as briefly depicted in Fig. 12.

### Surface modifications of PCL nanofibers

Hydrolysis and aminolysis procedures were performed to introduce oxygen-containing and amino groups on the surface of electrospun PCL nanofibers. The hydrolysis was conducted using NaOH solution in the concentrations of 0.5, 1, and 2 M and three incubation time points; 1, 6 and 12 h. At each incubation time point, the treated samples were brought out from the treatment solution, washed by deionized (DI) water several times and incubated in DI water overnight to remove the unreacted NaOH. Then, samples were dried at room temperature for 5 h and following that were dried out overnight under vacuum at 30 °C.

The aminolysis process was carried out using the hexamethylenediamine (HMD)/isopropanol (IPA) solutions that were prepared in the concentrations of 0.5, 1, and 2 M. The nanofibrous samples were submerged and incubated within the aminolysis solution for 1, 12, and 24 h at 37 °C. After reaching the incubation time point, the samples were removed from the treatment solution, washed by DI water multiple times and incubated in DI water overnight to remove the remnant HMD from the surface. Subsequently, the samples were dried at room temperature for 5 h and placed in a vacuum drier at 30 °C overnight. Table 2 represents a detailed overview on the preparation of samples.

**Table 2:** The conditions applied for aminolysis and hydrolysis of PCL nanofibers.

Samples	Abbreviation	Treatment	
		Treatment agent	Incubation time (h)
Pristine PCL	PCL	–	–
Sample 1	S1	NaOH 0.5 M	1
Sample 2	S2	NaOH 0.5 M	6
Sample 3	S3	NaOH 0.5 M	12
Sample 4	S4	NaOH 1 M	1
Sample 5	S5	NaOH 1 M	6
Sample 6	S6	NaOH 1 M	12
Sample 7	S7	NaOH 2 M	1
Sample 8	S8	NaOH 2 M	6
Sample 9	S9	NaOH 2 M	12
Sample 10	S10	HMD/IPA 0.5 M	1
Sample 11	S11	HMD/IPA 0.5 M	12
Sample 12	S12	HMD/IPA 0.5 M	24
Sample 13	S13	HMD/IPA 1 M	1
Sample 14	S14	HMD/IPA 1 M	12
Sample 15	S15	HMD/IPA 1 M	24
Sample 16	S16	HMD/IPA 2 M	1
Sample 17	S17	HMD/IPA 2 M	12
Sample 18	S18	HMD/IPA 2 M	24

### Characterizations

#### Structural and morphological evaluations

The structural properties of nanofibrous mats and morphology of the nanofibers were evaluated using a QUANTA FEG 450 Field Emission Scanning Electron Microscope (FEI, USA). In order to preparation of the specimens, the mats were punched, sputter-coated with a thin layer (8 nm) of gold (DST3, Iran), and micrographed at 20 kV accelerating voltage. The diameter of PCL nanofibers was measured using Image J software (1.47v, National Institute of Health, USA) and reported by averaging at least 100 individual nanofibers for each sample.

#### Elemental analysis

Elemental analyses of the PCL-based samples were conducted through energy dispersive X-ray spectroscopy (EDS). For this purpose, an Octane Elite Energy Dispersive X-ray Spectroscope (Gatan Inc., USA) were utilized which has been coupled with the FESEM device. The relative percentages of Carbon (C), Oxygen (O) and Nitrogen (N) were analyzed throughout the samples and reported in Table 1.

#### Surface wettability assessment

The wettability of as-prepared PCL-based samples was investigated using a static contact angle measurement device (IRASOL/CA-500A, Iran) at ambient temperature. The process of absorbing the droplet of water was recorded by a framing camera that was capable of capturing high-speed events. Afterwards, the contact angle measures between the water droplets and surface of samples were analyzed from the acquired images (at second 5) using the corresponding software.

#### Surface functional groups study

The potential alterations in the surface functional groups of samples were investigated using an Attenuated Total Reflectance Fourier Transform Infra-Red (ATR–FTIR) spectrometer (Tensor II/Bruker, Germany). The analyses were performed using transmittance mode in the wavenumber range of 4000–400 cm^−1^ and by spectral resolution of 2 cm^−1^. For each sample, the recorded data was acquired from the 32 scans.

#### Thermal analysis

The thermogravimetric analysis (TGA) of the samples was conducted using a Q600 thermogravimetric analyzer (TA Instruments, USA). Approximately 10 mg of the samples were weighed and placed into an aluminum pan to be heated from 24 to 600 °C by the heating rate of 10 °C min^−1^ under an inert atmosphere (Argon gas). Origin pro 9.1 software (Originlab Corporation, Northampton, MA, USA) was used to plot the derivative thermograms of the samples.

#### Crystallinity assessment

The crystallinity of the PCL-based samples was evaluated by the X-ray diffraction (XRD) technique. This characterization was performed using an Xpert instrument (Philips, The Netherlands). Monochromatic CuKα radiations (λ = 1.54056 Å), generated in the voltage and current of 40 kV and 40 mA respectively, were applied to collect the diffractrograms in step size of 0.08° s^−1^ and 2θ range of 10°–80°.

#### Mechanical testing

The mechanical behavior of the electrospun PCL samples was evaluated using a uniaxial tensile testing device (Santam, Karaj, Iran). Prior to the test, the nanofibrous mats were cut into the 40 mm × 10 mm rectangular-shaped strips, and their thicknesses were sharply measured via a micrometer. The as-prepared specimens were placed and fixed between inner sides of grips, covered by the specimen holders, and drawn at the crosshead rate of 5 mm/min up to the mechanical failure. The test was conducted 5 times for each sample and the data were reported as mean ± SD.

### In vitro studies

#### Cell viability tests

The viability and proliferation of cultured cells on the surface of PCL-based samples were investigated using the MTT assay evaluations (n = 5). The nanofibrous mats were cut into spherical pieces (6 mm diameter), put on the bottom of a 96-well plate, sterilized using UV light (for 30 min), washed with antibiotic-containing PBS (pH: 7.4), and enriched DMEM/F12 cell culture medium. The numbers of cultured L929 cells on the PCL-based samples were 2.5 × 10^4^, 1.5 × 10^4^ and 0.5 × 10^4^ for 24, 72, and 120 h time points respectively. By reaching each time point, multiple PBS washing steps were gently performed and subsequently the supernatant culture medium was replaced by the MTT solution (100 µl, 0.25 mg ml^−1^). The MTT solution was incubated on the samples for 4 h at 37 °C and atmosphere of 5.0% CO_2_. Afterwards, the MTT solution was removed and replaced by 100 µl of DMSO. The plate was wrapped in foil while placed on a shaking machine for 20 min to dissolve the formazan crystals. Afterward, a Microplate Spectrophotometer (Epoch, USA) was utilized to analyze the intensity of absorbance at 570 nm.

#### Cell attachment and morphology evaluations

The attachment, spreading and morphology of cultured L929 cells on the PCL-based samples were studied using Live/Dead and SEM cell adhesion assays. Prior to the Live/Dead assay, five specimens from the each opted sample were cut into the square pieces (1 × 1 cm) and prepared for the study. More precisely, the specimens were sterilized under UV light (30 min), separately placed on the bottom of the plate wells and washed several times using antibiotic-containing PBS (pH: 7.4) as well as cell culture medium. The test was conducted at three time points 24, 72, and 120 h. After reaching a time point, the supernatant cell culture medium was replaced by the freshly-prepared staining medium, comprised of propidium iodide (PI) and fluorescein diacetate (FDA), and incubated for 10 min. Afterwards, the stained samples were gently washed three times by PBS (pH: 7.4) and attachment, spreading and morphology of the cells were observed using an IX71 inverted fluorescence microscope (Olympus, Japan). The green- and red-stained cells in the micrographs were indicatives for the live and dead cells respectively.

The spreading morphology, cell-nanofiber interactions and potential infiltration of the L929 cells were studied through the SEM cell adhesion assay. For this test, chosen samples were prepared according to the method explained for Live/Dead assay. However, this study was performed merely in the time point which seemed to be optimal (72 h). By reaching the time point, the supernatant culture medium was removed and samples were gently washed by PBS (pH: 7.4) two times. Subsequently, samples were incubated with the fixation solution (Glutaraldehyde 2.5% in PBS) at 37 °C for 2 h. The fixative was then removed and samples were washed by PBS (pH: 7.4) three times to remove any remnant residue of glutaraldehyde. The samples were subsequently dehydrated in a gradient of ethanol solutions (30, 50, 70, 90 and 100%). The prepared samples were sputter-coated with a thin layer of gold (8 nm) and studied using field emission microscopy at the accelerating voltage of 20 kV.

### Statistical analysis

The single factorial analysis of variance (ANOVA) and Tukey’s multiple comparison tests of SPSS program, v.23 (IBM, Armonk, NY, USA) were administered to evaluate the statistical significance of the data. The results were reported as a mean ± standard deviation, and P < 0.05 was considered statistically significant.

## Supplementary Information

Supplementary Figures.

## Data Availability

The data that support the findings of this study are available from the corresponding author, Dr. Esmaeil Mirzaei, upon reasonable request.
