# Supplementary material for: Surface modification of polycaprolactone nanofibers through hydrolysis and aminolysis: a comparative study on structural characteristics, mechanical properties, and cellular performance
Source: Sci Rep. 2023 Jun 9;13:9434. doi: 10.1038/s41598-023-36563-w (PMC10256742; doi:10.1038/s41598-023-36563-w)
Supplement: Supplementary file 1 — Supplementary Figures. [file 41598_2023_36563_MOESM1_ESM.docx]

**Surface Modification of Polycaprolactone Nanofibers through Hydrolysis and Aminolysis Treatment: A Comparative Study on Structural Characteristics, Mechanical Properties, and Cellular Performance**

Razieh Yaseri^1^, Milad Fadaie^1^, Esmaeil Mirzaei^1*^, Hadi Samadian^3^, Alireza Ebrahiminezhad^4*^

^1^ Department of Medical Nanotechnology, School of Advanced Medical Sciences and Technologies, Shiraz University of Medical Sciences, Shiraz, Iran.

^2^ Nanomedicine and Nanobiology Research Center, Shiraz University of Medical Sciences, Shiraz, Iran

^3^ Pharmaceutical Sciences Research Center, Health Institute, Kermanshah University of Medical Sciences, Kermanshah, Iran.

^4^ Biotechnology Research Center, Shiraz University of Medical Sciences, Shiraz, Iran.

***Corresponding authors:**

E. Mirzaei: e_mirzaei@sums.ac.ir

A. Ebrahiminezhad: a_ebrahimi@sums.ac.ir

**Supplementary Data**


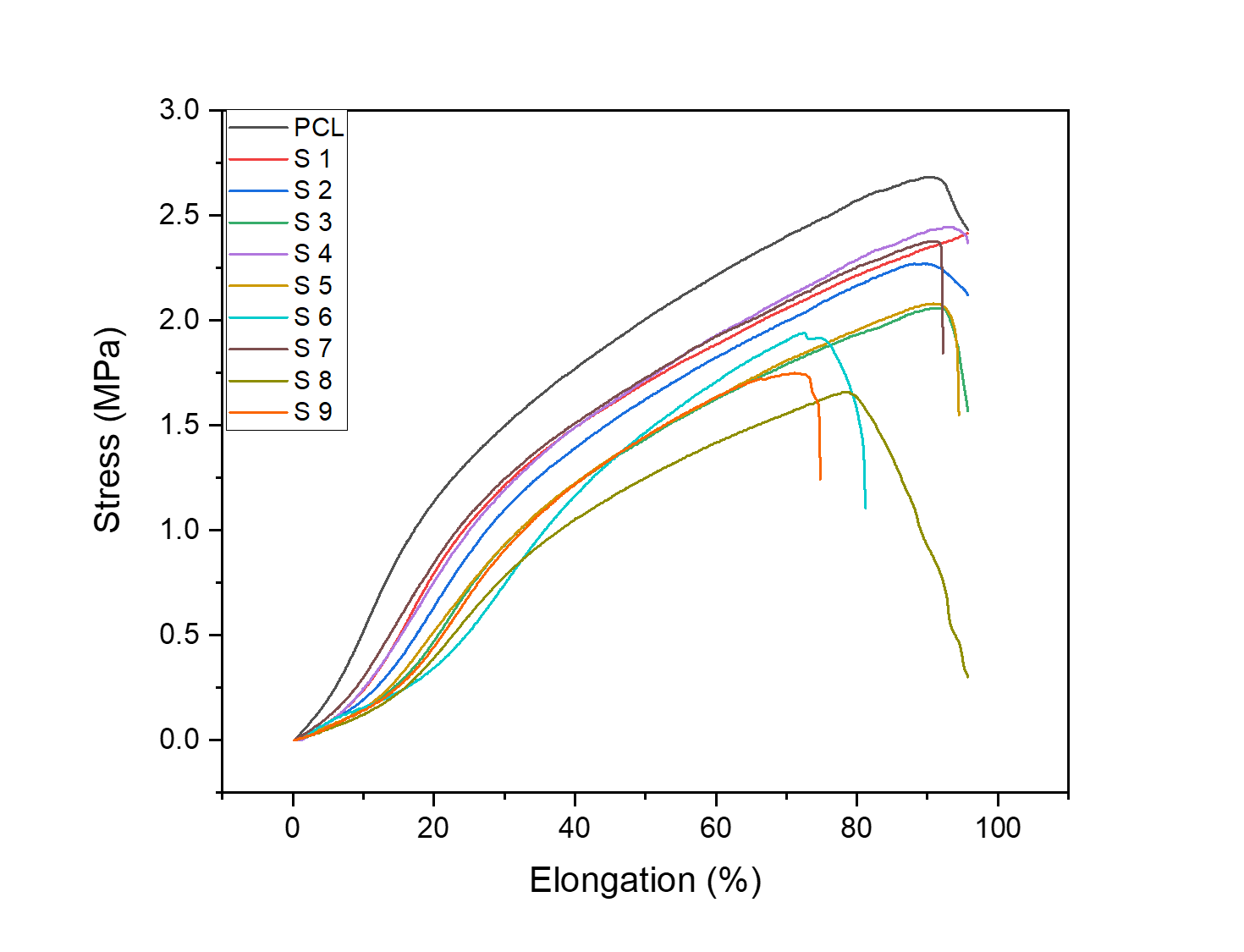


**Figure S1.** Mechanical graph of PCL and hydrolyzed samples


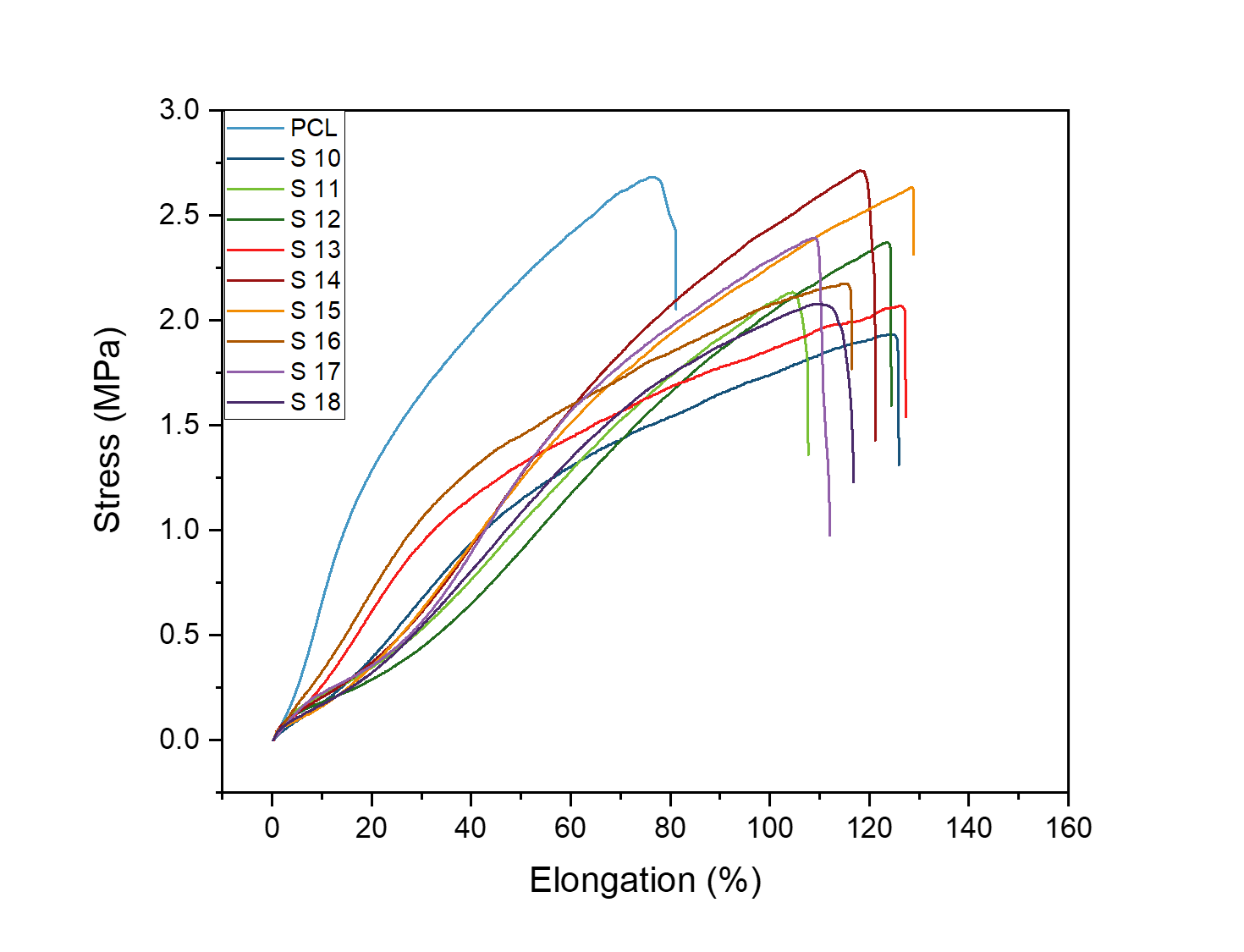


**Figure S2.** Mechanical graph of PCL and aminolyzed samples
